# Personalization of IVF-ICSI workflow based on patient characteristics improves IVF laboratory outcomes and embryo ploidy by PGT-A

**DOI:** 10.1186/s13048-022-01061-6

**Published:** 2022-12-01

**Authors:** Brandon A. Wyse, Noga Fuchs Weizman, Janice Montbriand, Rima Kharonsky, Ran Antes, Rina Abramov, Svetlana Madjunkova, Clifford L. Librach

**Affiliations:** 1 CReATe Fertility Centre, Toronto, ON Canada; 2 Racine IVF Unit, Lis Maternity Hospital, Tel Aviv Sourasky Medical Center, Affiliated to the Sackler Faculty of Medicine, Tel Aviv University, Tel Aviv, Israel; 3 Department of Anesthesia, Sunnybrook Health Sciences Centre, Toronto, ON Canada; 4 Department of Obstetrics and Gynecology, University of Toronto, Toronto, ON Canada; 5 Department of Physiology, University of Toronto, Toronto, ON Canada

**Keywords:** IVF-ICSI, IVF outcome, Denudation, Timing, Fertilization, PGT-A, Oocyte maturation, OPU, Trigger

## Abstract

**Background:**

Intracytoplasmic sperm injection (ICSI) has become a common method of fertilization in assisted reproduction worldwide. However, there are still gaps in knowledge of the ideal IVF-ICSI workflow including the optimal duration of time between induction of final oocyte maturation, oocyte denudation and ICSI. The aim of this study was to examine outcomes following different workflow protocols in IVF-ICSI procedures in blastocysts that have undergone undisturbed incubation and preimplantation genetic testing for aneuploidy (PGT-A) prior to transfer.

**Methods:**

Retrospective secondary analysis of 113 patients (179 IVF cycles, 713 embryos), all of whom have gone through IVF-ICSI and PGT-A using undisturbed culture. Predictive test variables were the length of time from: trigger to OPU, OPU to denudation, and denudation to ICSI. Outcome metrics assessed were: maturation, fertilization, blastulation and euploid rates. Generalized Estimated Equations Linear Model was used to examine the relationship between key elements of a given cycle and continuous outcomes and LOESS curves were used to determine the effect over time.

**Results:**

In a paired multi-regression analysis, where each patient served as its own control, delaying OPU in patients with unexplained infertility improved both maturation and blastulation rates (b = 29.7, *p* < 0.0001 and b = 9.1, *p* = 0.06, respectively). Longer incubation with cumulus cells (CCs) significantly correlated with improved ploidy rates among patients under 37, as well as among patients with unexplained infertility (*r* = 0.22 and 0.29, respectively), which was also evident in a multiple regression analysis (b = 6.73, *p* < 0.05), and in a paired analysis (b = 6.0, *p* < 0.05). Conversely, among patients with a leading infertility diagnosis of male factor, longer incubation of the denuded oocyte prior to ICSI resulted in a significantly higher euploid rate (b = 15.658, *p* < 0.0001).

**Conclusions:**

In this study we have demonstrated that different IVF-ICSI workflows affect patients differently, depending on their primary infertility diagnosis. Thus, ideally, the IVF-ICSI workflow should be tailored to the individual patient based on the primary infertility diagnosis. This study contributes to our understanding surrounding the impact of IVF laboratory procedures and highlights the importance of not only tracking “classic” IVF outcomes (maturation, fertilization, blastulation rates), but highlights the importance that these procedures have on the ploidy of the embryo.

**Supplementary Information:**

The online version contains supplementary material available at 10.1186/s13048-022-01061-6.

## Background

Intracytoplasmic sperm injection (ICSI) was first introduced in 1992 by Palermo et al. and has since become a common method of fertilization in assisted reproductive technology worldwide [1, 2]. However, there are still gaps in knowledge of the ideal IVF-ICSI workflow including the optimal duration of time between induction of final oocyte maturation, oocyte denudation and ICSI [3].

Current literature is divided, with some reports recommending extending incubation periods of oocytes following retrieval with their surrounding cumulus cells (CC) [4–6], while others have failed to observe potential benefits on any embryological or clinical outcomes [3, 7–15].

As for the optimal interval between denudation of the oocyte from its surrounding CC to the ICSI procedure, the literature is once again equivocal. While some have observed higher pregnancy rates [16], it has been postulated that beyond the optimal window, fertilization increases, but is accompanied by detrimental effects on developmental potential [17, 18]. This, in turn, could be attributed to in-vitro oocyte aging, hallmarked by detrimental cellular and molecular changes [19, 20]. In a systematic review by Wang et al. adverse effects of prolonged denudation of the oocytes were reported in 12 studies [15]. These in-vitro oocyte aging effects were seen more commonly in older women, thereby reducing the likelihood of clinical pregnancies [18, 20–22].

In general, most centers aspire to less than 41 h between triggering final oocyte maturation and ICSI, based on more conservative data available [23, 24]. Specifically, it has been suggested that for patients with repeat abnormal fertilization, oocytes should be denuded immediately after collection, followed by a careful and rapid ICSI to mitigate spontaneous oocyte activation-induced abnormal fertilization and possible aneuploidy [15].

In the current report, we aimed to examine the outcomes following different workflow protocols in IVF-ICSI procedures, on blastocysts that have undergone undisturbed incubation followed by preimplantation genetic testing for aneuploidy (PGT-A) prior to transfer.

## Results

### Baseline characteristics and overall cohort outcomes

A total of 113 patients undergoing 179 cycles were included in this analysis, producing a total of 713 embryos with overall maturation, fertilization, blastulation, and euploid rates of 75.4% ± 1.3, 81.7% ± 1.5, 63.4% ± 2.1, and 43.6% ± 2.4%, respectively. Patient characteristics and cycle parameters are presented in Table 1. In a stratified analysis blastulation rate was significantly higher in the non-ARA group 71.7% ± 3.0% compared to 52.1% ± 2.9% in the ARA group (*p* < 0.0001) as well as patients with male factor (75.4% ± 5.2% in the male factor group compared to 60.4% ± 4.2% in non-male factor group (*p* < 0.01)). Following stratification, the older population had a significantly lower proportion of euploid embryos (38.2% ± 3.3% compared with 53.0% ± 3.2% in the rest (*p* < 0.01)).

**Table 1:** Baseline characteristics of the study groups. The number of patients per group is indicated in the table heading. ARA – advanced reproductive age (>37yo), No ARA – not advanced reproductive age (<37yo)

	Overall ***n*** = 113	ARA ***n*** = 67	No ARA ***n*** = 46	Male Factor ***n*** = 11	Unexplained ***n*** = 35
**Oocyte Age (years)**	37.6 ± 3.6 (27–45)	39.8 ± 0.2 (37–45)	33.7 ± 0.2 (27–36)	32.8 ± 0.8 (27–36)	34.0 ± 0.3 (27–36)
**Sperm Age (years)**	39.6 ± 6.1 (26–60)	41.8 ± 0.6 (32–60)	36.2 ± 0.4 (26–44)	34.0 ± 1.2 (26–44)	37.1 ± 0.5 (31–44)
**BMI (kg/m**^**2**^**)**	24.1 ± 5.0 (14.8–52.8)	24.2 ± 0.4 (14.8–44.5)	24.1 ± 0.7 (16.7–52.8)	27.7 ± 1.7 (21.3–48.1)	23.6 ± 0.8 (16.7–52.8)
**AMH (pmol/L)**	12.3 ± 7.9 (0.5–40)	11.2 ± 0.8 (2–40)	13.7 ± 0.9 (0.5–40)	19.2 ± 1.6 (10.6–33.6)	13.3 ± 1.1 (0.5–40)
**Total Gonadotropin (IU)**	3801.9 ± 1065.4 (1050–6675)	3711.4 ± 110.0 (1050–6675)	3913.7 ± 105.1 (1950–6675)	3900.8 ± 201.9 (2400–4875)	3981.6 ± 140.1 (2400–6675)
**Estradiol on Trigger (pmol/L)**	8954.4 ± 6484.3 (1171–38,825)	7631.6 ± 494.9 (1171–32,335)	10,737.2 ± 883.4 (2108–38,825)	13,256.3 ± 2474.4 (4459–38,825)	10,523.7 ± 1103.0 (2108–29,778)
**Trigger to OPU (h)**	35.9 ± 0.8 (34.2–38.7)	36.0 ± 0.1 (34.8–38.7)	35.8 ± 0.1 (34.2–37.5)	36.0 ± 0.2 (35–37.5)	35.9 ± 0.1 (34.2–37.5)
**OPU to Denudation (h)**	1.4 ± 1.1 (0.1–5.5)	1.1 ± 0.1 (0.1–4.4)	1.7 ± 0.1 (0.3–5.5)	1.1 ± 0.2 (0.3–2.5)	1.9 ± 0.2 (0.3–5.5)
**Denudation to ICSI (h)**	1.2 ± 0.7 (0.1–5.2)	1.1 ± 0.1 (0.1–4.8)	1.2 ± 0.1 (0.1–5.2)	1.2 ± 0.2 (0.1–3.0)	1.2 ± 0.1 (0.5–2.2)

### Correlation analysis

**Fig. 1:**
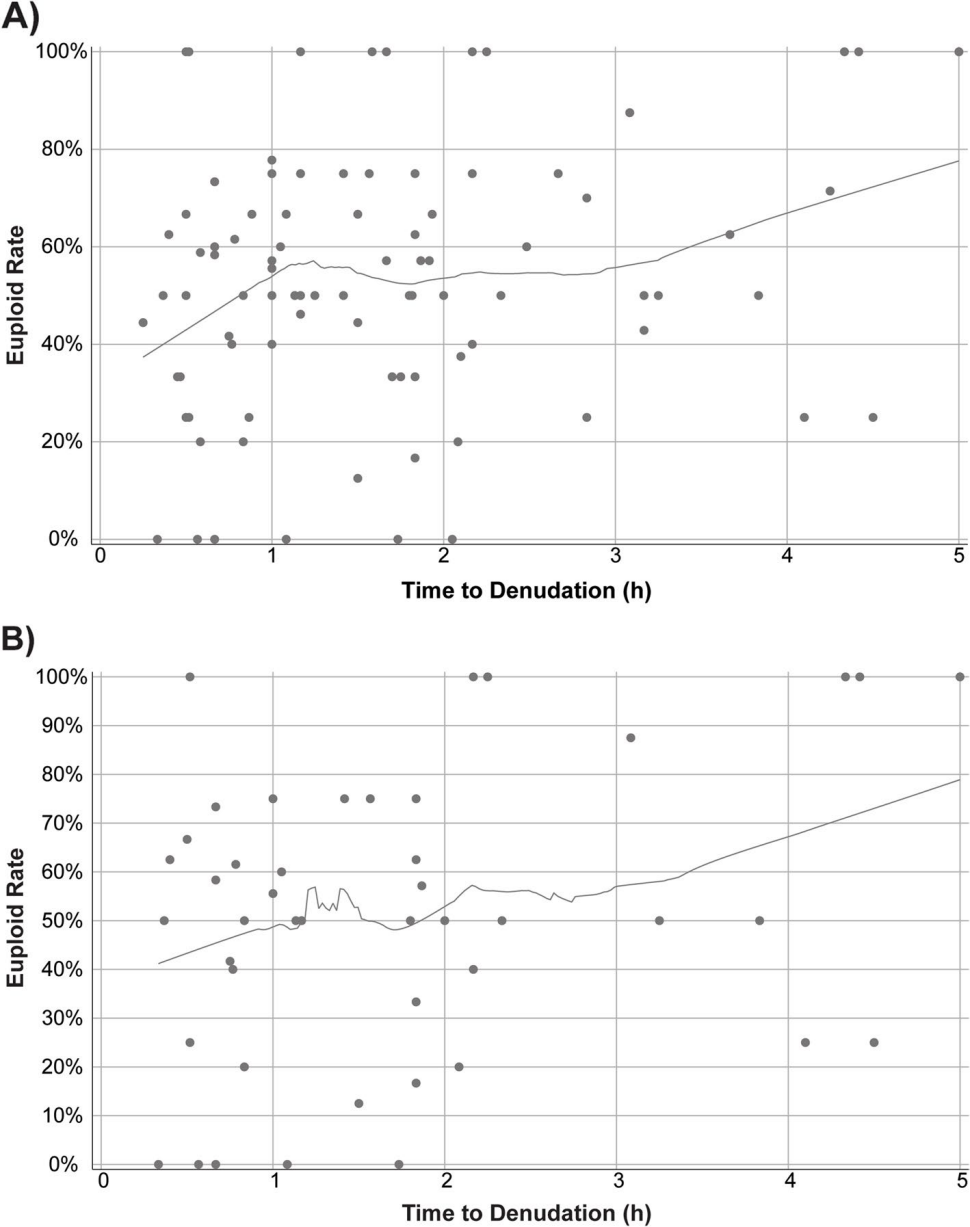
Locally estimated scatterplot smoothing (LOESS) correlations for time to denudation compared to embryo ploidy rate per IVF cycle in **A**) non-ARA group (*n* = 66) and **B**) unexplained group (*n* = 51)

When examining the duration of time from trigger administration to OPU, we did not observe a significant correlation with any of the measured outcomes in the overall cohort. Longer incubation with CCs significantly correlated with improved ploidy rates among the non-ARA group (under 37), as well as in the unexplained fertility group (Table 2). To determine the optimal time of incubation, LOESS analysis was conducted. In the non-ARA group, there was a 20% improvement in euploid rate when oocytes were incubated with their cumulus cells for at least 1 hour and up to 3 hours. After 3 hours, there is an observed additional 20% improvement from 3 to 5 hours of incubation, however, the low number of cycles at the 3–5 hour timepoints make this improvement non-significant (Fig. 1A). In the unexplained group, there was a 10% improvement in blastulation rate when oocytes were incubated with their cumulus cells for at least 1 hour, after which there was no further improvement observed with longer duration of incubation (Fig. 1B). In the male factor group (*n* = 15), delaying ICSI after denudation led to reduced blastulation, albeit with a higher euploid rate (Table 2). Finally, when examining the duration of time from trigger administration to ICSI (a sum of all timepoints collected in this study), there was no significant correlation with blastulation or euploid rate (data not shown).

**Table 2:** Correlation between laboratory procedure and outcome for all patients per IVF-ICSI cycle (*n* = 179). The number of cycles per group is indicated in the table heading. ARA – advanced reproductive age (>37yo), No ARA – not advanced reproductive age (<37yo)

		ARA (>37yo) ***n*** = 113		No ARA (27-36yo) ***n*** = 66		Male Factor ***n*** = 15		Unexplained ***n*** = 51	
		***r***	***P value***	***r***	***P value***	***r***	***P value***	***r***	***P value***
*OPU to Denudation*	*Maturation Rate*	–	–	–	–	–	–	–	–
	*Fertilization Rate*	–	–	–	–	–	–	–	–
	*Blastulation Rate*	–	–	–	–	–	–	–	–
	*Euploid Rate*	–	–	0.22	0.039	–	–	0.29	0.04
*Denudation to ICSI*	*Maturation Rate*	–	–	–	–	–	–	–	–
	*Fertilization Rate*	–	–	–	–	–	–	–	–
	*Blastulation Rate*	–	–	–	–	−0.61	0.016	–	–
	*Euploid Rate*	–	–	–	–	0.61	0.02	–	–

### Multi-regression analysis

In a multi-regression analysis, oocyte age was a significant negative predictor of euploid rate across all groups (Table 3). In the unexplained group, prolonged incubation of the oocytes with their surrounding cumulus led to a significant improvement of the euploid rate (b = 6.73, CI = 12.69–4.89, *p* < 0.05) (Table 3). Among patients with male factor, longer incubation of the denuded oocyte prior to ICSI, resulted in significantly higher euploid rate (b = 15.658, CI = 14.467–16.850, *p* < 0.0001).

**Table 3:** Cycle level multi-regression analysis for all IVF cycles (*n* = 179) from all patients in the study. The number of cycles per group is indicated in the table heading. ARA – advanced reproductive age (>37yo), No ARA – not advanced reproductive age (<37yo)

	ARA (>37yo) ***n*** = 113				No ARA (27-36yo) ***n*** = 66				Male Factor ***n*** = 15				Unexplained ***n*** = 51			
		***b***	***CI***	***p***		***b***	***CI***	***p***		***b***	***CI***	***p***		***b***	***CI***	***p***
*Blastulation Rate*	Oocyte Age	−3.370	−6.141 to −0.598	0.017	Sperm Age	1.625	0.405 to 2.846	0.009	Oocyte Age	−2.902	−4.090 to −1.714	< 0.0001	Sperm Age	3.434	3.065 to 3.802	< 0.0001
*Euploid Rate*	Oocyte Age	−4.709	−8.215 to − 1.261	0.007	Oocyte Age	−3.625	−5.975 to − 1.275	0.003	Denudation to ICSI	15.658	14.467 to 16.850	< 0.0001	OPU to Denudation	​6.73	4.89 to 12.69	0.027
									Oocyte Age	−3.230	−4.425 to −2.035	< 0.0001	Oocyte Age	−4.75	−7.78 to −1.71	0.002

### Paired cycle analysis

For better control of interpatient variability, we performed a paired analysis on all IVF-ICSI cycles. Only patients with more than one IVF-ICSI cycle during the study period were included to gain better understanding of the effects changes in IVF-ICSI workflow have on cycle outcomes. There were no significant predictors in the ARA and no-ARA groups, and we were under powered to observe differences in the MF group. However, in the unexplained group, delaying OPU was a strong positive predictor of maturation rate (b = 29.7, CI = 20.7–38.6, *p* < 0.0001) and blastulation rate (b = 9.1, CI = -0.5–18.7, *p* = 0.06). Furthermore, longer incubation of the oocytes with their surrounding CCs was a moderate positive predictor of euploid rate (b = 6.0, CI = 0.3–11.8, *p* < 0.05) (Table 4).

**Table 4:** Paired cycle level multi-regression analysis for patients who had multiple IVF cycles (*n* = 32)

	Unexplained ***n*** = 32			
		***b***	***CI***	***p***
*Maturation Rate*	LH	29.333	1.734 to 59.933	0.037
	Trigger to OPU	29.653	20.746 to 38.561	< 0.0001
*Fertilization Rate*	AMH	−0.592	−1.039 to −0.145	0.009
*Blastulation Rate*	Trigger to OPU	9.087	−0.494 to 18.667	0.06
*Euploid Rate*	Oocyte Age	−4.158	−7.074 to −1.242	0.005
	OPU to Denudation	6.045	0.256 to 11.835	0.041

## Discussion

The ideal workflow for IVF-ICSI has long been debated. Current guidelines are based on relatively outdated literature. In their systematic review, Wang et al. aimed at updating the current knowledge [15]. Most recent large series focused on either day 3 transfers [18], or a mixture of cleavage and blastocyst stage transfers [7]. Only one published series to date included embryos that have undergone PGT-A prior to transfer [3], and none examined the different workflow effects on embryos that undergo undisturbed incubation. In the current series, we aimed at including only blastocysts that have grown in an undisturbed incubation environment (i.e EmbryoScope™), followed by PGT-A prior to being transferred. Furthermore, following previous recommendations from the above series, we controlled for both paternal and maternal age, and examined the ploidy rate following differing workflows. In addition, we explored effects of different workflows on different patient populations, and finally, this series was designed to include a subset of patients with multiple cycles in which patients would serve as their own controls.

### Extending the lag between trigger of final oocyte maturation and oocyte retrieval to at least 37 h might be beneficial for patients with unexplained infertility

While extending the time between triggering final oocyte maturation and oocyte retrieval did not seem to have a beneficial effect in the overall cohort, it was a positive predictor of oocyte maturation and blastulation in patients with unexplained infertility (Table 4). A previous study by Deng et al. concluded that delaying OPU could be beneficial and suggested that this effect could be especially beneficial in patients with low ovarian reserve [25]. However, by virtue of their study design they were not able to differentiate between different effect sizes, in relation to the infertility diagnosis. In the current study we were able to stratify our analysis by infertility diagnosis, enabling us to show that patients with unexplained infertility would benefit most from this strategy. Importantly, we did not encounter any cases of ovulation prior to the retrieval, however, this should be validated in prospective cohorts. Though a previous study reported that extending this interval has the potential to improve live birth rates [26], our study was not designed to explore these effects.

### Longer incubation of oocytes with their surrounding CCs improved ploidy rates in patients with unexplained infertility

In this cohort, incubation of oocytes with their surrounding cumulus cells for at least 1 hour improved the euploid rate in patients with unexplained infertility (Table 2 and Fig. 1). This effect was sustained in a multi-regression analysis controlling for all possible covariates available (Table 3), and was evident in the paired analysis as well, where patients served as their own controls (Table 4). Notably, most previous studies did not see a positive effect following such elongated incubation, and most did not check for euploid rate [15]. In their study published in 2020, Carvallo et al. observed improved live birth rates following incubation of at least 4 hours in patients with MF and in patients who did not have any fertilizations following conventional IVF, however our study was not designed to examine such effects [27]. Finally, in a paired analysis, allowing for patients to serve as their own controls, lengthened incubation of the oocytes with their surrounding CCs was a positive predictor of euploid rate among the unexplained infertility group. To our knowledge, this is a novel finding which stems from our unique analysis methodology.

### Among patients with MF diagnosis, delaying ICSI improved ploidy

In the MF cohort, delaying ICSI by increasing the incubation of the denuded oocyte resulted in more euploid blastocysts, reiterating findings by Falcone et al. [16]. However, this may not necessarily translate to improved live birth rates as there was a negative correlation with time to ICSI and blastulation rate (Table 2), resulting in fewer blastocysts to transfer. This finding should be further assessed with larger studies. Previous studies have cited increased fertilization following longer denudation times, at the cost of detrimental effects on developmental potential, hallmarked by detrimental cellular and molecular changes [19, 20]. To our knowledge, ours was the first study to assess the effect of these changes on euploid rates and with the increasing adoption of PGT-A worldwide, decreasing the time to pregnancy is no longer driven by the number of blastocysts obtained, but the number of euploid blastocysts obtained, so delaying ICSI at least in MF patients, may in a large enough cohort, decrease the time to pregnancy.

## Conclusions

It is critical to have an accurate diagnosis for the cause for infertility, and to take this cause into account during IVF laboratory procedures. In this study we have demonstrated that different IVF-ICSI workflows affect patients differently depending on their infertility diagnosis. Patients with unexplained infertility may benefit from increased time lag between the trigger and the OPU. The younger patient population, and patients without egg factor may benefit from longer incubation of oocytes with their surrounding cumulus cells. Finally, patients with MF diagnosis may benefit from delaying ICSI despite the oocytes being denuded for longer time periods. Thus, our data suggests that the ideal workflow should be tailored to the infertility diagnosis. This study contributes to our understanding surrounding the impact of IVF laboratory procedures. It highlights the importance of not only tracking “classic” IVF outcomes (maturation, fertilization, blastulation rates), but highlights the impact that these procedures have on the embryo ploidy. This study is limited by the study design, namely the patient population was skewed to patients who were undergoing IVF due to advanced reproductive age. The study was not designed to determine the underlying mechanism of these findings. Future randomized prospective studies with larger sample sizes are needed to validate these findings.

## Methods

### Ethics approval

This study was approved by Veritas Independent Review Board (IRB approval #16447).

### Study design

A secondary analysis of patients participating in a prospective trial, all of whom have gone through IVF-ICSI and PGT-A using undisturbed culture (i.e. EmbryoScope™), between February 2020–February 2021, at CReATe Fertility Centre (Toronto, ON, Canada). Patients with PCOS and/or endometriosis were excluded from the study.

### Data collection

Patient characteristics including age, body mass index (BMI), fertility diagnosis, pre-stimulation blood work (AMH, LH, and estradiol levels on day 2/3), stimulation characteristics (gonadotropin dose, trigger medication, and estradiol levels on trigger day), and sperm parameters (paternal age and total motile sperm) were used as covariates in the analysis. Outcome variables were collected including IVF laboratory outcomes (number of retrieved oocytes, number of mature oocytes (MII), number of fertilized oocytes (2PN), number of blastocysts, and number of euploid embryos. Different metrics were calculated based on the above collected data, including maturation rate (number of mature oocytes divided by number of retrieved oocytes), fertilization rate (number of 2PN divided by number of mature oocytes), blastulation rate (number of blastocysts divided by number of 2PN) and euploid rate (number of euploid blastocysts divided by total number of blastocysts). Finally, the predictive tested variables that were collected included the length of time from trigger injection to oocyte pick-up (trigger to OPU), the length of time cumulus-oocyte complexes were incubated following OPU (OPU to denudation) and the length of time the oocytes were incubated for following denudation of the cumulus cells prior to ICSI (denudation to ICSI). The length of time for each predictive variable not randomized and was mainly driven by the clinical workload in the IVF.

### Statistical analysis

Subgroups were made for key groups of interest, based on primary infertility diagnosis. The most common diagnosis was advanced reproductive age (ARA - > 37 years old) (*n* = 113), followed by unexplained infertility (*n* = 35) and male factor (*n* = 11). See Supplemental Fig. S1 for the study flowchart. Analysis was conducted on the full study cohort, and where sample size allowed, analyses were carried out both in the overall sample and by subgroups. Outliers were identified over the entire set using standardized scores and were removed during analyses.

Generalized Estimated Equations (GEE; Linear Model) was used to examine the relationship between key elements of a given cycle and continuous outcomes (maturation, fertilization, blastulation, and euploid rates). An auto-regressive correlation structure was used. Significance was defined as *p* < 0.05 for all GEE analysis. The individual cycles were identified, and analyses were run at the cycle level. Variables were entered in a univariate GEE (Linear, Autoregressive correlation structure). Significant variables (*p* < 0.05) and trends (*p* = 0.06) were entered into a multivariate linear GEE predicting the relevant continuous outcomes.

### LOESS curves

To take a closer look at how timing (e.g OPU to denudation) may affect outcomes over time, LOESS (locally estimated scatterplot smoothing) curves with 50% smoothing were created. Curves were generated when a significant or trending correlation (positive or negative) was seen between a timing predictor and a key outcome in the univariate analysis. These were undertaken in key subgroups (ARA, male factor and unexplained).

## Supplementary Information

**Additional file 1: Supplemental Figure S1.** Study flowchart.

## Data Availability

The datasets used and/or analyzed during the current study are available from the corresponding author on reasonable request.
