# Supplementary figures and images for: Personalization of IVF-ICSI workflow based on patient characteristics improves IVF laboratory outcomes and embryo ploidy by PGT-A
Source: J Ovarian Res. 2022 Dec 1;15:124. doi: 10.1186/s13048-022-01061-6 (PMC9714092; doi:10.1186/s13048-022-01061-6)

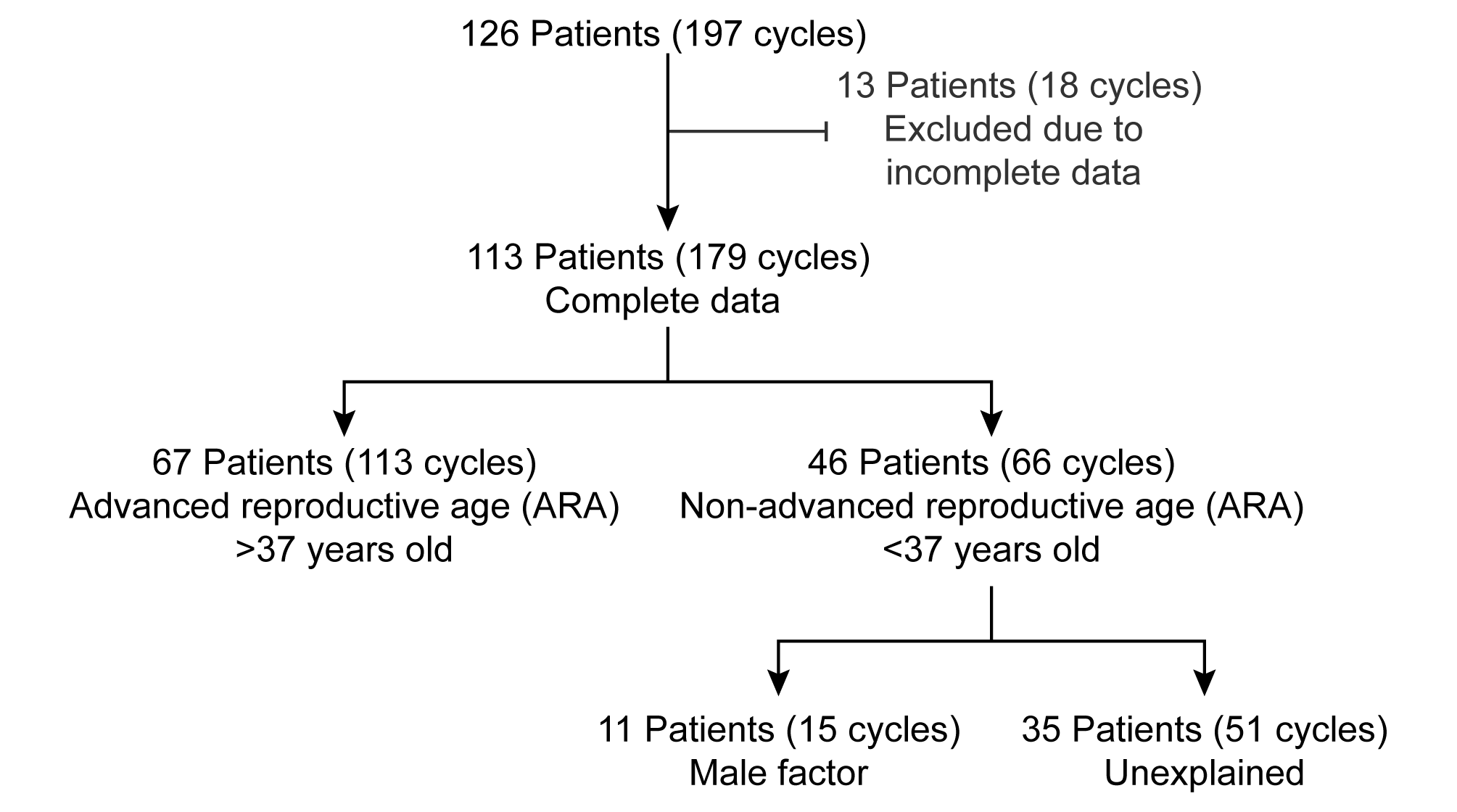

Supplement: Supplementary file 1 — Additional file 1: Supplemental Figure S1. Study flowchart. [file 13048_2022_1061_MOESM1_ESM.tif]
